# Molecular Tools for Targeted Control of Nerve Cell Electrical Activity. Part I

**DOI:** 10.32607/actanaturae.11414

**Published:** 2021

**Authors:** D. V. Kolesov, E. L. Sokolinskaya, K. A. Lukyanov, A. M. Bogdanov

**Affiliations:** Shemyakin-Ovchinnikov Institute of Bioorganic Chemistry, Moscow, 117997 Russia

**Keywords:** optogenetics, chemogenetics, thermogenetics, action potential, membrane voltage, neurointerface, ion channels, rhodopsin, chemoreceptors, GPCR, neuronal activity stimulation, neuronal excitation, neuronal inhibition

## Abstract

In modern life sciences, the issue of a specific, exogenously directed manipulation of a cell’s biochemistry is a highly topical one. In the case of electrically excitable cells, the aim of the manipulation is to control the cells’ electrical activity, with the result being either excitation with subsequent generation of an action potential or inhibition and suppression of the excitatory currents. The techniques of electrical activity stimulation are of particular significance in tackling the most challenging basic problem: figuring out how the nervous system of higher multicellular organisms functions. At this juncture, when neuroscience is gradually abandoning the reductionist approach in favor of the direct investigation of complex neuronal systems, minimally invasive methods for brain tissue stimulation are becoming the basic element in the toolbox of those involved in the field. In this review, we describe three approaches that are based on the delivery of exogenous, genetically encoded molecules sensitive to external stimuli into the nervous tissue. These approaches include optogenetics (Part I) as well as chemogenetics and thermogenetics (Part II), which are significantly different not only in the nature of the stimuli and structure of the appropriate effector proteins, but also in the details of experimental applications. The latter circumstance is an indication that these are rather complementary than competing techniques.

## INTRODUCTION

Deciphering the principles of the nervous system functioning in higher multicellular organisms is a fundamental problem in neuroscience. For many decades, the traditional approach to its solution has been reductionism; i.e., extrapolation of the results observed in simple model systems to complex neuronal assemblies that cannot be directly analyzed (e.g., mammalian brain). The numerous disadvantages of such an approach and the emergence of revolutionary techniques for imaging and stimulation of cellular processes have pushed neuroscientists to look for ways to directly investigate the entire organizational nomenclature of the nervous system and the complex biological phenomena associated with its functioning.

Today, minimally invasive methods for a selective stimulation of the activity of nerve cells and brain structures are among the major tools used in neuroscience. Here, we describe the main ones: optogenetics (the first part of this review), chemogenetics and thermogenetics (the second part), with an emphasis on the nature, physicochemical properties, and principles for developing effector molecules that mediate cellular stimulation and are used in biochemical and neurobiological experiments. We will also focus on the molecular mechanisms underlying the functioning of these genetically encoded tools.

The review focuses on the key characteristics of the described approaches (spatial and temporal resolution, toxicity, invasiveness, etc.), provides a comparative analysis of these characteristics in relation to the topical problems of modern neuroscience, and discusses the prospects for improving these neurostimulation tools.

## OPTOGENETICS

Optogenetics is a group of techniques that use visible light to control the functional activity of cells by means of light-sensitive proteins whose genes are introduced into the biological system in advance (for a detailed review, see [1, 2, 3, 4, 5, 6, 7]). Light is not only the primary energy source for anabolic processes in the entire biota, but also the most important physical stimulus playing a key role in the physiology and biochemistry of the representatives of all living kingdoms. During evolution, a rich repertoire of light-sensitive molecules has emerged. They differ in their physical and biochemical properties, structure, and functions [8, 9, 10, 11, 12, 13, 14]. This circumstance provides the prerequisites for the use of a wide range of genetically encoded effector molecules in optogenetics to affect a wide variety of biochemical targets [2, 3, 7].

Before the advent of optogenetic tools, chemical compounds with photolabile bonds were used to mediate light-driven effects on a cell physiology. Such photoeffectors, which include photoactivatable amino acids, oligonucleotides, and compounds for a light-dependent release of other molecules, have been engineered in abundance and remained in use until now, developing independently of the genetically encoded tools [15, 16, 17].

**Optogenetics in molecular biology**

In molecular biology, the optogenetic approach is primarily used for the control and manipulation of protein–protein interactions [2, 18, 19]. In this case, effector molecules are natural proteins or individual domains whose oligomeric state or interaction with other proteins changes upon absorption of light: e.g., phytochromes, bacteriophytochromes, and cryptochromes.

Phytochromes are plant photoreceptors containing a covalently bound tetrapyrrole chromophore that is sensitive in the red region of the spectrum [18, 20]. The optogenetic use of these proteins is primarily based on the natural light-dependent reversible interaction between phytochrome PhyB and the transcription factors PIF3 and PIF6, and the most striking examples are the systems for optical control of Gal4 transcription factor activity [21], protein splicing activation [22] in yeast cells, and rapid reversible translocation of Rho family GTPase activators to the plasma membrane of mammalian cells [23]. Cryptochromes are FAD-containing, blue and violet sensitive photoreceptors found in all cellular life forms, which are also capable of photodimerization with partner proteins. In particular, photodimerization of the plant cryptochrome CRY2 with the transcription factor CIB1 [24, 25, 26, 27] was used to demonstrate light-dependent DNA recombination [28] and to control the epigenetic status of chromatin [29] in mammalian cells. There are reports on the use of the CRY/CIB system for controlling transcription in yeast [30] and the activity of the phosphoinositide metabolism in COS-7 cells with a high spatial resolution [31]. The light-sensitive PHR domains of CRY2 were used to develop tools for controlling the release of intracellular calcium [32], including those operating in single T-cells* in vivo *[33].

The three-dimensional conformation of some photoproteins can change significantly in response to light absorption [2, 18, 19]. In optogenetics, this property is used to manipulate molecular targets. A striking example is light-oxygen-voltage (LOV) proteins from a large family of light-sensitive flavoproteins found in plant, fungal, and bacterial cells [34, 35, 36]. LOV domains have been used to develop dozens of optogenetic techniques [2, 18]; e.g., control of gene expression [37, 38], modulation of enzymatic activity [39] and signaling involving cyclic nucleotides [40], regulation of genome editing [41], and photosensitization [42].

BLUF (blue-light sensors using flavin-adenine dinucleotide) family flavoproteins, which are mainly of bacterial origin, similarly to LOV-domains, undergo photoactivation accompanied by structural rearrangements [43, 44, 45, 46, 47]. Optogenetic applications of these flavoproteins include the PICCORO transcription activation system [48] and photoactivation of adenylate cyclases [49, 50] and guanylate cyclases [51].

A separate group of optogenetic effectors is constituted by UVR8 photoreceptors that absorb in the UV range owing to their intrinsic tryptophan residues and are involved in photoprotective reactions in plants [52]. In plant cells, UVR8 homodimers dissociate in response to ultraviolet light irradiation and monomers bind to the E3-ubiquitin ligase COP1 [52, 53, 54, 55, 56]. There are reports on the use of this protein for targeted regulation of transcription [19, 57, 58] and control of intracellular transport of proteins and their secretion [59]. Optogenetic control of transcription also uses prokaryotic proteins of the xanthopsin family [60, 61], which carry a covalently bound *p*-coumaric acid chromophore and have an unusual photocycle [62].

The reversible light-induced interaction of the bacterial phytochrome BphP1 and its natural partner protein PpsR2 form the basis of another platform for optogenetic experiments using bacterial proteins [63]. The unique characteristics of the BphP1–PpsR2 system include its activation in the near-IR wavelength range (740–780 nm), ability to use endogenous biliverdin of eukaryotes, including mammals, as a chromophore, and spectral compatibility with blue light-based optogenetic systems [63]. Further studies of the system led to the designing of its updated version, where the Q-PAS protein, produced using genetic engineering methods, is used instead of natural PpsR2 as a BphP1 partner [64]. The Q-PAS-based system has no limitations related to the PpsR2 properties, such as a large size, multidomain structure, and tendency to oligomerize [64].

The system based on the bacterial phytochrome BphP1 was also used for optogenetic control of the activity of receptor tyrosine kinases [65]. For this purpose, the catalytic domain of the tropomyosin kinases TrkA and TrkB, which are present on the cell membrane as inactivated dimers, was fused with a photosensitive core of BphP1. BphP1 dimerization under illumination with far red (640–680 nm) and near-IR (740–780 nm) light activated the kinase dimer and enabled light-driven reversible modulation of the enzyme activity [65].

Green fluorescent protein (GFP) family members are widely used as fully genetically encoded fluorescent probes. In addition, there are several examples of the use of GFP-like proteins in optogenetics. For example, the reversibly switchable Dronpa protein was found to simultaneously change its fluorescent properties and oligomeric state: it monomerizes after exposure to blue light [66]. This property was used for light-dependent induction of the activity of target proteins (e.g., protein kinases) flanked at the N- and C-termini by Dronpa monomers and inactive in the dark due to steric blocking by a fluorescent protein dimer [66, 67]. Another example is the engineering of a photocleavable protein based on mMaple [68] that is characterized by irreversible photoconversion from a green to red fluorescent state. Although this photoconversion is accompanied by a cleavage of the polypeptide chain before the chromophore, two parts of the protein remain tightly bound through many non-covalent interactions. There is a permuted mMaple variant, called PhoCl (PhotoCleavable) [69], which spontaneously dissociates into two parts after exposure to 405-nm light. PhoCl was used to design the proteins with light-induced activity: Cre recombinase, Gal4 transcription factor, HCVp viral protease, and photocleavable cadherin to study the transfer of mechanical tension between cells [69, 70].

A separate area of optogenetics is the use of phototoxic proteins: i.e., proteins that produce significant amounts of reactive oxygen species (ROS) in response to irradiation with light [71, 72]. The most popular objects are the phototoxic proteins KillerRed (GFP-like red fluorescent protein) and miniSOG (LOV-based flavin- binding protein), as well as their mutated variants [42, 73, 74, 75]. The advantages of such genetically encoded photosensitizers (in comparison with conventional chemical ones) include the possibility to guide them toward any cell compartments and subcompartments using protein localization signals and, at the level of the organism, to target cell populations using tissue-specific or inducible promoters. Local ROS production enables targeted manipulation of biological systems: e.g., inactivating target proteins [73, 76], triggering various pathways of cell death [77, 78, 79], damaging genomic DNA [80], and destroying target cells in model organisms [81, 82, 83].

Protein engineering is widely used in the design of optogenetic systems [2, 18], which makes it possible not only to integrate effector molecules into the context of target intracellular interactions, but also to adapt their activity to a particular experimental task. This adaptation may be exemplified by the optobody, an optogenetically activated intracellular antibody (intrabody, iB) built on the basis of modified LOV domains (namely, the so-called Magnets, chimeric variants of the Vivid photoreceptor which are capable of light-dependent heterodimerization [84]) and anti-GFP nanobody fragments [85]. A composite optogenetic tool based on recombinant iBs was used for reversible regulation of the activity of endogenous proteins in mammalian cells [86]. The activity of endogenous actin and RAS GTPase can be manipulated by guiding effectors of two optogenetic systems (BphP1-Q-PAS, which is sensitive to near IR light, and LOV, which is activated by blue light) with a fluorescently labeled iB [86].

According to their molecular mechanism, there are two groups of optogenetic manipulations in molecular biology: allosteric manipulations, where the photosensitive domain affects enzymatic activity or access to the substrate binding site, and dimerization-based manipulations: i.e., those associated with a light-dependent change in the oligomeric status of effector domains, which affects the activity of target proteins comprising the chimeric molecule. Combinations of the two approaches are also possible [18]. As we have illustrated above, such indirect involvement of optical effectors comes handy in a wide range of model systems, but it is not typical of neurobiological optogenetics. The activity of electrically excitable cells is controlled by effector molecules that directly affect the physiological status of cells.

**Optogenetics in neurobiology**

The activity of electrically excitable cells is closely related to the electrical potential on their plasma membrane [87]. The potential is generated, in particular, thanks to the activity of voltage-gated selective ion channels; i.e., channels that allow passage of certain ions at a certain level of membrane polarization [87]. The transmembrane gradient of ions, for which voltage-gated channels are selective (primarily Na^+^, K^+^, Cl^-^), causes a short-term shift of the membrane potential, termed the action potential. When the membrane is depolarized below the threshold level or is hyperpolarized, the arising current rapidly decays or integrates with other ionic currents, which can, depending on the direction of integrated currents, initiate or, on the contrary, prevent the generation of a new action potential. Therefore, by changing transmembrane ionic currents and the ratio of ion concentrations inside and outside the cell, it becomes possible to control the functional activity of cells using various ionic transporters.

The first report on an instance of activation of neurons by light dates back to 1971, when laser light was found to nonspecifically stimulate nerve cells in tissues of the mollusk Aplysia [88]. The ability of genetically encoded effector molecules to influence transmembrane ionic currents upon light activation was first observed during heterologous expression of bacteriorhodopsin in *Xenopus laevis *oocytes [89]. The same system was used to demonstrate the induction of photocurrents upon expression of channelrhodopsin 1 (channelopsin-1) [90], a retinal-containing proton channel from the single-cell green alga *Chlamydomonas reinhardtii*. It is noteworthy that this photoreceptor, which has a high homology with bacteriorhodopsins, plays a role in the phototaxis of algal cells [91]. Later, channelrhodopsin 2 (ChR2) from *C. reinhardtii *was functionally expressed in mammalian cells and its activity as a light-dependent cationic channel capable of depolarizing the cell membrane was described [92]. One of the first examples of use of an optogenetic tool for stimulating neurons was associated with the expression of rhodopsin from *Drosophila *in a primary culture of rat neurons [93]. But in this case, the minimum set of transgenes that ensured the activity of the effector consisted of three coding sequences (rhodopsin, arrestin-2, and the α-subunit of the G-protein), the latency of the stimulation ranged from hundreds of milliseconds to seconds, and addition of a retinal solution to the cells was required in the experiment. Finally, control of neuronal activity using single-component optogenetic effectors based on channelrhodopsin 2 (ChR2) was shown almost simultaneously in four studies [94, 95, 96, 97]. From a methodological point of view, these studies form the basis of modern neurobiological optogenetics. It is noteworthy that due to the efficiency of channelrhodopsin, yet early experiments could use complex model systems, in particular to control the behavior of the *Caenorhabditis elegans *nematode [96] and partially restore the visual sensitivity of transgenic mice with degenerative retinal disorders [97]. These pioneering works reported a high spatial and temporal resolution of activation: stimulation on a millisecond time scale [94] or at frequencies of up to 20 Hz [96], and the possibility of targeted manipulation of fine subcellular neuronal structures.

**Effector molecules**

**Fig. 1:**
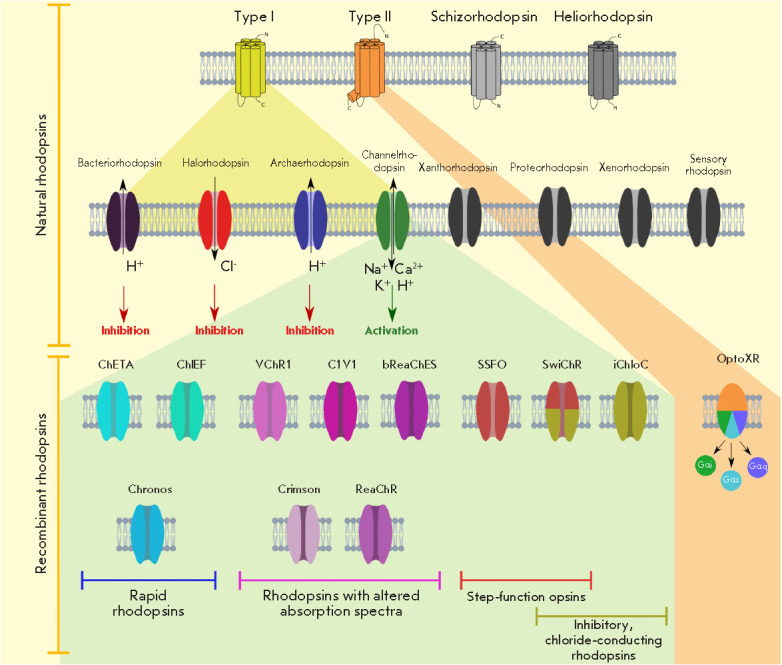
The diversity of rhodopsins and their use in optogenetics. The top row depicts the four largest families of natural rhodopsins. The second row from the top presents the main groups of microbial rhodopsins. The next row presents chimeric channelrhodopsins (left) and type 2 rhodopsin-derived molecules (right) optimized for performing special optogenetic tasks. In the top two rows, families/types of rhodopsins that have not yet been used in optogenetic applications are shown in gray; those involved in optogenetics are represented by spectral colors. Chimeric molecules are differentiated by colors depending on their functional features (the color legend is described in the lower part of the figure)

Thuswise, rhodopsins constitute the major class of effector molecules in optogenetics of electrically excitable cells [1, 3, 98, 99] (the diversity of rhodopsins is illustrated in *Fig. 1*). These light-sensitive transmembrane proteins bear a retinal-based chromophore that, as a protonated Schiff base, is covalently (via a lysine residue) attached to the seventh transmembrane helix of the protein backbone [100, 101]. Rhodopsins form two independent families: microbial rhodopsins (type 1 rhodopsins) and animal rhodopsins (type 2 rhodopsins). Despite their structural similarity, representatives of these two rhodopsin types are characterized by an extremely low homology of amino acid sequences, apparently arising independently during convergent evolution [102]. Type 2 rhodopsins are known primarily as visual pigments that are specifically expressed in the cells (rods) of the animal retina; however, the proteins of this family are involved in other physiological processes, both associated and not associated with photoreception [100, 102]. The mechanism of signal transduction during photoreception is an important distinguishing feature of type 2 rhodopsins. For example, the functional cycle of visual (rod) rhodopsin involves at least three cytoplasmic proteins: G-protein transducin, rhodopsin kinase, and arrestin. This circumstance complicates the use of animal rhodopsins in heterologous systems and thereby reduces their value as optogenetic effectors. Microbial rhodopsins are found in archaea, bacteria, eukaryotic microorganisms (algae and fungi), and even giant viruses [100, 101, 102, 103, 104, 105]. The molecules of this family perform a wide range of functions associated with photosensitivity: light-dependent enzymatic activity, photoreception, and ion transport [100, 103, 106]. According to their working principle, rhodopsins involved in ion transport are, in turn, subdivided into ion pumps and channels. It is ion-transporting rhodopsins, which are capable of generating currents in the cell membrane and changing its polarization, that are used in optogenetics as effectors (*Fig. 1*). Among wild-type microbial rhodopsins, these include bacteriorhodopsins, proton pumps that pump these cations out of the cell; halorhodopsins, chloride pumps that transport Cl– into the cell; and channelrhodopsins that are non-selective cation channels allowing passage of H^+^, Na^+^, K+, and Ca^2+^ ions through the membrane [3, 107, 108]. The proteins of the first two groups, upon photoactivation (by green and yellow light, respectively), cause membrane hyperpolarization, which in the case of electrically excitable cells leads to inhibition of the action potential, thereby acting as inhibitory effectors [107] *(Fig. 1*). Channelrhodopsins absorbing blue light, on the contrary, depolarize the membrane and promote the stimulation of nerve cells. Determination of channelrhodopsins’ spatial structure [109] has enabled the application of rational design principles to the development of chimeric variants of these proteins and the switch from cationic to anionic selectivity of the ionic pore [110, 111], expanding the repertoire of optogenetic inhibitors. Later, natural chloride anion-conducting channelrhodopsins were also discovered [112]. In addition to the abovementioned inhibitory channelrhodopsins, a rich palette of artificial channelrhodopsins optimized for solving particular optogenetic tasks has been developed using protein engineering methods. These include: fast channelrhodopsins (e.g., ChETA, ChIEF, Chronos) that provide, in particular, high-frequency (up to 200 Hz) stimulation of neurons [1, 113, 114, 115]; the so-called step-function opsins [116] that have a significantly increased inactivation time and are therefore able to maintain a corresponding transmembrane current for a relatively long time at a short duration of the light stimulus (there are both variants causing membrane depolarization [117]; and inhibitory hyperpolarizing variants [110]).

Wild-type channelrhodopsins are activated by blue light, which has a small penetration depth in animal tissue and can be toxic to neurons. In addition, blue light excites most of the existing fluorescent calcium ion indicators that can be used in conjunction with optogenetic tools. In this regard, a number of spectrally optimized variants of channelrhodopsins with absorption maxima shifted to the red region have been developed (these include VChR1, C1V1, Chrimson, ReaChR, etc.) [1, 114, 117, 118, 119, 120] (*Fig. 1*). Rhodopsins with artificially altered cationic permeability are represented, in particular, by calcium-translocating channelrhodopsin (CatCh) that preferentially conducts Ca^2+^ ions and is in demand in studies of calcium signaling [121]. In addition, unique rhodopsins, Na^+^ pumps, were found in marine bacteria [122], and they were used to develop selective transporters of potassium, rubidium, and cesium cations [123, 124]. Recently, an elegant method for a genetically engineered modification of a ChR2 mutant was proposed, which led to inverted topology of the insertion of this protein into the cell membrane and its conversion from an activator into an inhibitor upon photoactivation [125, 126].

The last few years have been full of discoveries of new groups and even families of rhodopsins which can be considered as promising optogenetic tools. For example, channelrhodopsins *Gt*_CCR1–4 from the flagellate unicellular alga *Guillardia theta*, which are light-sensitive cationic channels, proved structurally closer to the rhodopsins of haloarchaea than to classical ChR2 [106, 127, 128]. Recently, *Gt*_CCR4, which has activation/inactivation kinetics similar to those of ChR2, was shown to have a significantly higher photosensitivity, as well as higher selectivity for sodium cations [106, 129]. In 2018, a new rhodopsin family, heliorhodopsins, was discovered using functional metagenomics methods [103]. These proteins, like type 1 rhodopsins, bind retinal in the all-*trans* conformation and are abundant in archaea, bacteria, microalgae, and their viruses. Data on the spatial structure of heliorhodopsins [130, 131] confirm their structural homology with bacteriorhodopsins and an unusual, inverted compared to other rhodopsins, orientation in the membrane (with cytoplasmic Nand extracellular C-termini, *Fig. 1*). The biological function of these pigments is still unknown, but the inability of heliorhodopsins to transfer ions and their relatively slow (on a second scale) photocycle is evidence pointing to their photoreceptor role [103]. The availability of high-resolution structural data provides hope that, in the near future, heliorhodopsins may become an object of protein engineering aimed, in particular, at optimizing their molecules for the needs of optogenetics. Representatives of two families of light-dependent proton pumps, xenorhodopsins [132] and schizorhodopsins [133], may also become optogenetic actuators. Interestingly, the proteins of both families pump protons into the cell, which distinguishes them from the previously described bacterio- and archaerhodopsins, which transport H^+^ in the opposite direction.

Finally, chimeric photosensitive G-protein-coupled receptors (Opto GPCRs), such as optoXR, constitute a distinctive class of optogenetic tools. These molecules are built on the basis of type 2 rhodopsins (visual rhodopsins of animals), in which the intracellular loops of rhodopsin are replaced by loops from, e.g., adrenergic or dopamine receptors [134, 135]. In this case, photostimulation of rhodopsin can initiate various intracellular signaling cascades, depending on the type of receptor donating intracellular loop regions (*Fig. 1*) [136, 137, 138, 139]. Detailed information about Opto GPCR studies can be found in a dedicated review [5].

The biophysical properties of the rhodopsins used in optogenetics have been studied in detail [100, 140, 141]. For example, the three-dimensional structures of channelrhodopsins from *C. reinhardtii *have been resolved [109, 142] and the photocycle of microbial rhodopsins has been investigated not only by time-resolved spectroscopy [100], but also by time-resolved X-ray diffraction analysis [143, 144] (their detailed description is beyond the scope of this review). However, it is worth mentioning two facts that are of fundamental value for the optogenetic use of microbial rhodopsins: (i) all type 1 rhodopsins use the all-*trans* retinal stereoisomer as a chromophore. The successful development of the so-called single-component (i.e., using an effector encoded by a single transgene) optogenetics is largely related to the presence of a sufficient amount of endogenous retinal in the nerve tissues of vertebrates, which excludes addition of this cofactor from the outside [145]; (ii) during the photocycle, retinal is photoisomerized into the 13-*cis*-conformation and then, remaining covalently bound to the protein backbone, spontaneously returns to its initial all-*trans *state [108]. This process lacks a dissociation stage, which enables multiple usage of the effector molecule, while its timescale – 10–20 ms – ensures a high temporal resolution of optical stimulation.

**Optogenetic experiment**

According to the key researchers involved in the implementation of neurobiological optogenetics, about the first 5 years of its development were devoted to the design and refinement of optogenetic experimental techniques [3]. In addition to the selection of successful photoeffector molecules (see the previous section), the delivery of a transgene to the target model system and the design features of an experimental setup play an important role in the matter. Here, we will briefly discuss these aspects.

Generally, strategies for the delivery and introduction of the genetic material of rhodopsin effectors may be reduced either to a transient expression in specific populations of nerve cells using viral vectors carrying rhodopsin genes [3] or to a stable expression of these genes in the brain of transgenic animals [3, 146, 147, 148]. In the former case, viral particles are usually injected into the animal’s brain. Early optogenetic studies gave preference to retroviral vectors. Modern studies usually use high titers of adeno-associated viruses (AAVs), whose genome sequences are often optimized to ensure a high expression level in specific types of brain cells [1]. In the last few years, modified rabies viruses have been used for the so-called retrograde (i.e., directed into the bodies of presynaptic neurons) targeted expression of rhodopsins [149, 150]. To increase the selectivity of “labeling” during heterologous expression of rhodopsins, promoters specific to a certain cell type [1, 3] (e.g., the hypocretin promoter (Hcrt) [151]) are used. In experiments on live embryonic brain slices, the transgene can be delivered using *in utero *electroporation; while in the body of transgenic animals, rhodopsin is expressed from birth. An increase in the specificity of optogenetic stimulation, which is effective in both the transient and stable expression of rhodopsins, can be provided by genetic manipulations using site-specific recombination [1, 3]. For example, Cre or Flp recombinases, which can be delivered to the brain by a separate vector or be stably expressed in the cells of transgenic animals, allow for highly selective turning on/off of the expression of a photoeffector gene in the studied cell populations [152].

The tissue and cell specificity of optogenetics as applied to the stimulation of the intact brain of experimental animals is provided by a combination of the genetic approach (specific targeted expression) and instrumental solutions for precision optical exposure. For example, light is delivered to the brain by means of a fiber-optic cable fixed to the animal’s skull, through an implanted optical cannula. The fiber-optic neurointerface is one of the key technological solutions that ensure success of the optogenetic approach [151, 153, 154]. The most important invention in the field of neurointerfaces for freely moving animals is autonomous wireless implants [155, 156, 157].

An essential aspect of the experiments on the optical manipulation of neuronal activity is the control of stimulation outputs at the level of individual cells and cell populations. Along with classical approaches to the direct monitoring of electrical activity (e.g., patch-clamp), which are often of limited applicability in stimulating an intact brain, fluorescent methods, such as imaging of genetically encoded calcium and voltage indicators, can be used [1, 3]. According to some authors, optogenetic tools include not only photoeffector molecules, but also fluorescent probes for neuroscience [158, 159]. The concept of combining optical stimulation and the monitoring of neuronal activity within one experiment, or all-optical electrophysiology, has been developed [4, 158, 160].

**Modern applications**

**Fig. 2:**
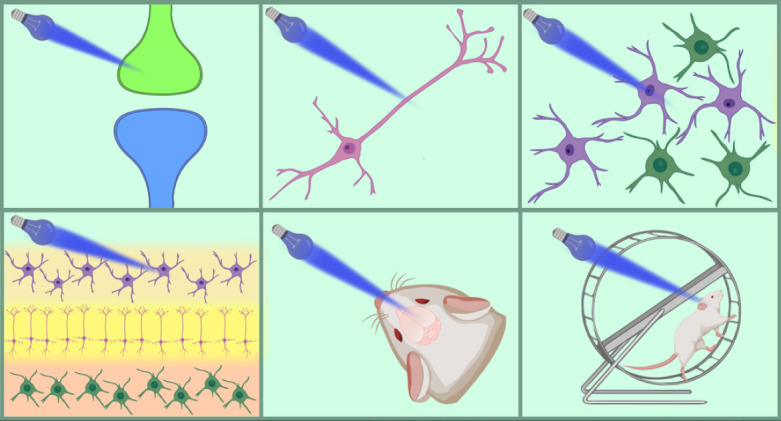
Optogenetics applications at different levels of the nervous system organization. The figure illustrates rhodopsin photoactivation in (left to right): a synaptic axon terminal; a single neuron *in cellulo*; a neuronal population *in cellulo*; a fresh brain tissue slice *ex vivo*; and the brain of a live and freely moving mouse *in vivo*. Adapted from [159]

A unique feature of the optogenetic approach is its versatile character and applicability in model systems of varying complexity (*Fig. 2*). This approach is used to investigate all levels of nervous system organization: in a culture of neurons *in cellulo*, live brain slices* ex vivo*, and the whole brain *in vivo *(in particular, awake, freely moving mammals) [159, 161]. Molecules mediating optical stimulation can be delivered to most highly specialized cells of the nervous system and their subcellular compartments, and the functional parameters induced by optogenetic stimulation range from the electrical activity of a single excitable cell to higher behavioral functions of mammals, such as learning, memory, etc.

Optogenetic tools have allowed neuroscientists to control the activity of neurons and neuroglial cells with high temporal and spatial resolution. This advantage of the method is especially important when studying* in vivo *tissue physiology and animal behavior. The resolution typical of optogenetic tools could not previously have been achieved using other neurobiological methods, such as deep brain stimulation (DBS) or administration of various drugs. The emergence of optogenetic methods in the arsenal of neuroscientists has enabled significant progress in understanding the formation and functioning of neural networks and signaling pathways in the mammalian brain [1, 3, 162]. They have been used to identify causal relationships between cellular activity and functional response, in particular, in experiments on a relationship between the activity of neural networks and the specific behavior of animals [163] and gain new information about various behavioral patterns in health and disease [164, 165].

Small rodents (mice and rats) are the main model objects in neurobiological research involving optogenetic tools. There are hundreds of studies on neuronal ensembles, networks, rhythmic brain activity, transmission, memorizing, and storage of information in the brain, learning, synaptic plasticity, neurogenesis, regulation of motor activity, hunger and thirst, sleep and wakefulness, sensory organs, biological rhythms, respiratory activities, and social behavior of these animals [1, 3, 6, 148, 164, 166, 167]. The optogenetic toolbox is also used to explore the neurobiology of fish [168], birds [169], and primates [170, 171]. Of course, the use of microbial rhodopsins in medicine and human neurophysiology research is of particular interest. Here, there are several closely related research areas: the study of the mechanisms of neurodegenerative diseases (Alzheimer’s disease [172, 173] and Parkinson’s disease [174, 175], epilepsy [176], etc.), mental disorders, and heart diseases in animal models and human neurons, finding approaches to the diagnosis of these pathologies using collected data, and screening of compounds potentially suitable for their therapy [3, 158]. Also, approaches to the therapeutic use of optogenetic tools are being developed. Currently, two clinical trials in the field of gene therapy for vision recovery using channelrhodopsins are being carried out in the U.S. [177]. Therapy for epilepsy [176] and hearing impairment [178] is coming soon.

**Method limitations**

Paradoxically, it is the extraordinary diversity and efficacy of the optogenetic approach that prompts researchers to pay significant attention to its shortcomings and limitations. In this case, we are dealing with a tool that has become a *de facto *standard for dozens of research areas, and its issues should thus draw more attention than the theoretical downsides in exotic techniques which can be reproduced by only a few laboratories in the world.

Below, we list the most significant problems associated with single-component optogenetics:

• Expression of microbial rhodopsins has limited applicability when working with invertebrates. As already mentioned, mammalian neurons contain a sufficient amount of retinal for inclusion in heterologically expressed rhodopsins, but in models, such as* Drosophila *or *Caenorhabditis*, at minimum addition of retinal to the diet of experimental animals is required [3].

• The spectral repertoire of microbial rhodopsins (at least, if activating and inhibiting molecules are considered separately) is rather poor. Even new variants of channelrhodopsins with absorption maxima shifted to the red region have a large spectral overlap with wild-type pigments. Although the use of several effectors with different activation profiles enables selective simulation of separate neuronal populations in the brain [179], this opportunity is rarely used in practice.

• Overexpression of microbial rhodopsins in nervous tissue can negatively affect the physiology of neurons [180], and their activation by blue light is potentially phototoxic.

• An obvious limitation of the method is the need to use complex fiber-optic devices fixed on the skull of animals. Methods of brain tissue irradiation without a special interface [181, 182] are less effective and have not yet become widespread [3].

• Finally, the intensity of optogenetic stimulation (both excitation and inhibition of neurons) cannot always be precisely controlled, and it can spill beyond the physiological limits. The problem is complicated by the heterogeneity of effector expression and light energy distribution in brain tissue, while precise stimulation is completely impossible in the depth of the tissue [159].

In the second part of this review, we will acquaint the reader with alternative approaches to specific neurostimulation: thermogenetics and chemogenetics.

## Abbreviations

AAV: adeno-associated virus
BLUF: blue-light sensors using flavin-adenine dinucleotide
ChR: channelrhodopsin
CIB1: cryptochrome interacting BHLH 1
COP1: coat protein complex 1
DBS: deep brain stimulation
FAD: flavin adenine dinucleotide
GFP: green fluorescent protein
IR: infra red
LOV: light-oxygen-voltage
PhoCl: photocleavable
PHR: photolyase homology related domain
PICCORO: PIxD complex dependent control of transcription
PIF: phytochrome-interacting factor
ROS: reactive oxygen species
UVR8: UV-B resistance 8 protein
